# Osteological, Biomolecular and Geochemical Examination of an Early Anglo-Saxon Case of Lepromatous Leprosy

**DOI:** 10.1371/journal.pone.0124282

**Published:** 2015-05-13

**Authors:** Sarah A. Inskip, G. Michael Taylor, Sonia R. Zakrzewski, Simon A. Mays, Alistair W. G. Pike, Gareth Llewellyn, Christopher M. Williams, Oona Y-C Lee, Houdini H. T. Wu, David E. Minnikin, Gurdyal S. Besra, Graham R. Stewart

**Affiliations:** 1 Faculteit Archaeologie, Universiteit Leiden, 2311 BE, Leiden, The Netherlands; 2 Department of Microbial and Cellular Sciences, School of Biosciences and Medicine, University of Surrey, Guildford, GU2 7TE, United Kingdom; 3 Department of Archaeology, University of Southampton, Avenue Campus, Highfield, Southampton, SO17 1BF, United Kingdom; 4 Ancient Monuments Laboratory, English Heritage Centre for Archaeology, Fort Cumberland, Fort Cumberland Road, Eastney, Portsmouth PO4 9LD, United Kingdom; 5 EPSRC National Mass Spectrometry Facility, Institute of Mass Spectrometry, College of Medicine, Swansea University, Swansea, SA2 8PP, United Kingdom; 6 Institute of Microbiology and Infection, School of Biosciences, University of Birmingham, Edgbaston, Birmingham, B15 2TT, United Kingdom; Hebrew University, ISRAEL

## Abstract

We have examined a 5th to 6th century inhumation from Great Chesterford, Essex, UK. The incomplete remains are those of a young male, aged around 21–35 years at death. The remains show osteological evidence of lepromatous leprosy (LL) and this was confirmed by lipid biomarker analysis and ancient DNA (aDNA) analysis, which provided evidence for both multi-copy and single copy loci from the *Mycobacterium leprae* genome. Genotyping showed the strain belonged to the 3I lineage, but the Great Chesterford isolate appeared to be ancestral to 3I strains found in later medieval cases in southern Britain and also continental Europe. While a number of contemporaneous cases exist, at present, this case of leprosy is the earliest radiocarbon dated case in Britain confirmed by both aDNA and lipid biomarkers. Importantly, Strontium and Oxygen isotope analysis suggest that the individual is likely to have originated from outside Britain. This potentially sheds light on the origins of the strain in Britain and its subsequent spread to other parts of the world, including the Americas where the 3I lineage of *M*. *leprae* is still found in some southern states of America.

## Introduction

Leprosy, caused by *Mycobacterium leprae*, has been described in historical texts since ancient times and archaeological and osteological findings indicate its existence in human populations for thousands of years [1]. There is scant detail concerning the origin of leprosy in European populations, but an epidemic of infection peaked between the 12^th^ and 14^th^ centuries. We and others have studied the genomes of medieval *M*. *leprae* strains to reveal that the decline of leprosy was not a result of changes in the pathogen genome [2–4], and so may have been due to other suggested factors such as changing socioeconomic conditions or interaction with concomitant epidemics of other infectious diseases such as tuberculosis and plague. By using a multidisciplinary approach applied to bioarchaeological remains, we reason it should be possible to understand more about the rise and decline of leprosy in Europe.

Burial GC96, the subject of the current study, was excavated from the Anglo-Saxon cemetery located to the northwest of Great Chesterford, on the boundary between Cambridgeshire and Essex. The excavation dates to the period between October 1953 and April 1954 and was from the second of three brief periods of rescue archaeology undertaken by Vera Evison on behalf of the Inspectorate of Ancient Monuments in the wake of commercial gravel extraction in the area [5]. Radiocarbon dating undertaken for the present study shows this burial dates to between the 5^th^ and 6^th^ centuries AD.

The skeleton shows some lesions suggestive of lepromatous leprosy (LL). The remains are amongst the earliest known in Britain with indications of leprosy. Although other cases of similar period are known in Britain [6–8], none of these earlier cases have been studied using modern techniques. Consequently, nothing is known of the causative strains of *M*. *leprae* at this time or of the likely geographical origin of the affected individuals. We have therefore applied osteological, biomolecular and geochemical isotopic analyses to GC96 in an attempt to understand the pathogen genome as well as the origin and any possible migrations undertaken by this individual and how this relates to the spread of the disease during this period.

## Methods

### 1. Grave description and Osteology

As excavations took place over 60 years ago, no permits were required for the described study, which complied with all relevant regulations. As the skeletal material is over 100 years old, it is not subject to the Human Tissue Act (2004). The original excavation was carried out with the permission of the landowner and tenant at the behest of the Inspectorate for Ancient Monuments [5]. The individual studied was specimen GC96 which is currently curated at the Department of Archaeology at the University of Southampton, UK.

**Fig 1:**
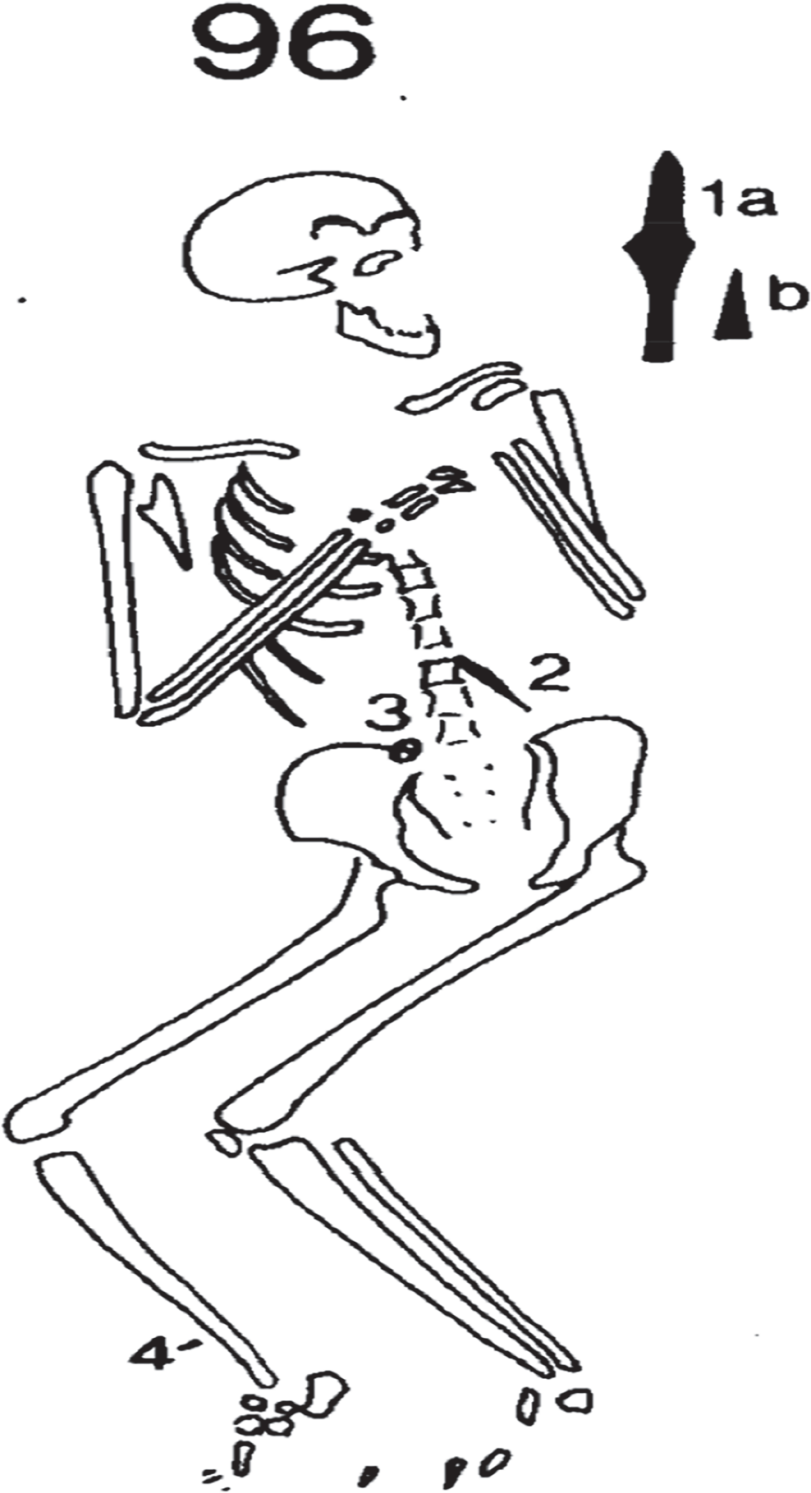
Grave sketch of inhumation 96 showing positioning of the skeleton and location of grave goods. 1 a *Spearhead*, angular, split socket with transverse rivet. Length 27.4 cm. b *Conical ferrule*, two decorative grooves. Length 10.2 cm. a and b were found before the grave was identified, about 8cm above floor of grave to left of skull. 2 *Knife*, slender, type 1. Length 12cm. Position: Point up under vertebrae at left waist. 3 *Oval buckle loop and tongue*. Transverse ribbing on the loop visible near the tongue. Diameter 2.5 cm. Position: Top right of pelvis. 4 *Bronze shoelace tag*, flat band folded and riveted together, two decorative lines. Length 2.1 cm. Position: Above right ankle. Reprinted from [5] under a CC BY license, with permission from the Council for British Archaeology, original copyright 1994.

Grave 96 was orientated west-east, with the head at the west end of the grave. The body was in a semi-flexed supine position at a depth of 91cm. The legs were flexed and the knees were pointing towards the right side of the grave. The arms were flexed at the elbow and the hands were placed on the left thorax/shoulder. The face was superiorly orientated with a lean toward the left of the grave (see Fig 1). The individual had a number of grave items including a spear and a conical ferrule, found above the left shoulder, a buckle loop and a knife in the abdominal area and shoelace tag found near the right ankle.

Bone preservation varies between good and excellent, with most of the cortical bone available for macroscopic inspection (75%+). The skull has suffered *post-mortem* damage to the facial area. There was no evidence for animal gnawing, but all of the bones have some minor root etching.

While the mandible and most of the vault is present, the facial bones are largely absent. With the exception of the left arm (humerus, radius and ulna), all of the major long bones are present. Only the left fibula is missing its distal epiphysis, the other bones were complete. The clavicles, *os coxae*, sacrum and scapulae are represented. Along with eight metatarsals (5 left and 3 right), all of the right tarsals and the left talus are present. Four proximal, one intermediate and one distal foot phalanges are present. The hand bones, pre-sacral vertebrae and ribs are missing.

Estimation of age was undertaken using epiphyseal fusion as outlined in Scheuer and Black (2000) [9] and dental wear as described in Brothwell (1981) [10]. Sex was determined from the sexually dimorphic traits of the cranium and the pelvis [11]. Alterations due to disease were recorded using careful visual examination of the remains.

### 2. Biomolecular methods

#### a). Ancient DNA analysis

1. Samples. The skeletal elements studied and bone weights sampled from these are shown in Table 1. An initial screening for evidence of *M*. *leprae* DNA was conducted on samples taken from the first metatarsal, left talus and fibula. Additional samples from the left talus, calcaneum and fibulae were used to assess reproducibility and to provide sufficient material for full genotyping.
2. DNA extraction. A guanidine thiocyanate (GuSCN)/silica-based kit (NucliSens, bioMérieux, Basingstoke, Hampshire, UK) was used to prepare DNA extracts for PCR testing according to the manufacturers’ instructions with additional modifications previously described [12]. The weights of bone powder tested are given in Table 1.
3. *M*. *leprae* screening methods. Three PCR methods were used to screen for evidence of remnant *M*. *leprae* genomic DNA. Two were for the multicopy RLEP element. One of these, a real-time PCR (RT-PCR) method using an intercalating fluorescent dye (EVAGreen) has been previously reported [12]. A second method for RLEP, employing a dual labelled hybridisation probe was developed in the course of this study. This method was designed to amplify template sizes of 78 bp and above, and should provide greater scope for detecting the pathogen from extremely degraded samples as well as improved specificity. The details of primers and probe are shown in Table 2. Additionally, we used a second probe method, for the 18-kDa-antigen gene, present in single copy in the *M*. *leprae* genome. Samples which were also positive using this method were taken forward for genotyping.
4. *M*. *leprae* genotyping methods. Positive bone extracts were subjected to SNP analysis using PCR methods which target the phylogenetically informative loci described by Monot and colleagues [13,14]. The primer sequences and nucleotide positions of corresponding SNPs are listed in Table S5 of Supplementary Information in reference [14]. Additionally we also used those primers described by Truman and co-workers [15] for further sub-typing 3I strains. These allow determination of the 4 main SNP types and also the subtype (A-O) of the strain. Primer sequences and assay conditions for many of these methods have been reported [2]. Therefore, Table 2 lists only new methods developed for genotyping or refinements to the earlier methods. Similarly, VNTR profiling of three variable loci, AGA(20), GTA(9) and 21–3 was undertaken as previously described (12).
5. Screening for *Mycobacterium tuberculosis* DNA in GC96 was undertaken using a dual-labelled hydrolysis probe method for the IS*1081* repetitive element [16].
6. PCR amplification details. PCR was performed in a final volume of 25μl, using a hot start Taq kit from Qiagen (product 203445). The reactions contained 25 pmol of forward and reverse primers, each in 1μl, 12.5 μl of the kit master mix, 2.5 μl non-acetylated bovine serum albumin (BSA, Sigma B4287) and 1μl of template. The kit magnesium ion concentration of 1.5 mM per reaction was supplemented to 2 mM for PCR methods using EVAGreen and to 3 mM MgCl_2_ for RT PCR with the RLEP and 18-kD hydrolysis probes. The probes were used at a final concentration of 100 nM. The volumes were made up to 25 μl with molecular biology grade water (Sigma). After an initial activation step of 14 min at 95°C, 41 cycles of amplification were performed on an Mx3005P RT-PCR platform (Agilent Technologies).
  The thermal profile of the amplification cycles consisted of denaturation at 95°C for 10 s, annealing (range 52–60°C) for 30 s and extension at 72°C for 30 s. Fluorescence data was acquired during the extension step in RT-PCR runs. Melt analyses was performed automatically at the end of runs monitored with EVAGreen and dissociation curves studied to identify likely positives.
7. Gel electrophoresis and automated DNA sequencing. PCR products were also run out on either 3% or 3.5% agarose gels in a TAE buffer system alongside appropriate DNA size markers (100 bp or 50 bp DNA ladders, Promega) to confirm product identity. Positive samples for SNP or MLVA typing were bulk purified on 3% (wt/vol) low-melting-point agarose (Invitrogen); bands were excised and purified using a Geneclean DNA isolation kit (Cat.No.1001-200, mpbio.com). Templates were sequenced using both forward and reverse primers by Beckman Coulter Genomics Ltd., Takeley, Essex, UK.

**Table 1:** An overview of the bone samples studied and the PCR findings.

Skeletalelement	Bonepowder(mg)	RLEP 111bpamplicon	RLEP 78bpanplicon	18-kDantigen 114bpamplicon	IS1081Probe	Mycolicacidanalysis
1^st^metatarsal(reactivebone)	70	-	-	-	ND	+
L Fibula 1	60	+	ND	+	-	+
L Talus 1	50	+	ND	+	-	+
Fibulae 2	50	+	+	+	ND	ND
L Talus 2	50	+	+	+	ND	ND
Calcaneum	100	+	+	±	ND	ND

ND = not determined. + = PCR positive. - = PCR negative. bp = base pair.

**Table 2:** Oligonucleotide primer and probe sequences for novel aDNA methods applied to burial GC96.

PCR locus	Primers	Probe sequence	Amplicon (bp)	Anneal temp.
RLEP F3	5’-gctggtatcggtgtcggcgg-3’	6[FAM]ttgaccggccctcagccagcaagcaggcat[BHQ1]	78	53
RLEP R3	5’-cacgatactgctgcacccggc-3’	6[FAM]ttgaccggccctcagccagcaagcaggcat[BHQ1]	78	53
18-kD F	5’-ctaatcgactgttgtttgcgcaac-3	[Joe]ctgcggtcaaaagcccgtcttagccatg [BHQ1]	114	56
18-kD R	5'-gccagcaaccgaaatgttcgga-3	[Joe]ctgcggtcaaaagcccgtcttagccatg [BHQ1]	114	56

#### b). Lipid biomarker analyses

Lipid biomarkers from GC96 were extracted, derivatised and fractionated, as described previously [17,18]. The extraction procedure was applied to dried bone powder already pre-extracted for DNA fragments: talus (51.6mg), metatarsal (62.6mg) and fibula (73.7mg). Standard biomass of *M*. *leprae* was available from a previous study [19]. In brief, the samples (Table 1) were hydrolysed by heating with 30% potassium hydroxide in methanol (2 ml) and toluene (1 ml) at 100°C overnight [17,18]. Standard biomass from *M*. *tuberculosis* and *M*. *leprae* were processed in parallel. Long-chain compounds were extracted as described previously [17,18] and the extract was treated with pentafluorobenzyl bromide (PFB) under phase-transfer conditions to convert acidic components into PFB esters. Subsequent separation was performed on an Alltech 209250 (500 mg) normal phase silica gel cartridge, which yielded separate fractions possibly containing non-hydroxylated PFB esters and mycolic acid (MA) PFB esters.

The MA PFB esters were reacted with pyrenebutyric acid (PBA) to produce PBA-PFB MA derivatives, which were refined on an Alltech 205250 (500 mg) C18 reverse phase cartridge [18]. The presence of PBA-PFB mycolates was investigated by reverse phase HPLC and the total MAs were collected and analysed by normal phase HPLC. Fractions collected from the normal phase separation were studied by further reverse phase HPLC [2,17].

The non-hydroxylated PFB esters were fractionated on an Alltech 205250 (500mg) reverse phase silica gel cartridge, using a water-methanol/methanol/methanol-toluene elution sequence [18]. A fraction enriched in mycocerosic acid and other longer chain PFB esters (>C20) was eluted by 100% methanol, with the more usual C12 to C20 esters eluting in the earlier water/methanol fractions. The fractions containing possible mycocerosates were analysed by negative ion chemical ionization gas chromatography mass spectrometry (NICI-GCMS), essentially as previously described [18]. Instrumental details are provided in S1 Fig. PFB esters, on NICI-GCMS, fragment to produce negative carboxylate [M – H]^-^ ions, which can be detected at high sensitivity. Selected ion monitoring (SIM) was used to search for mycocerosate carboxylate ions at *m/z* 367.6311 (C_24_), 395.6844 (C_26_), 409.7111 (C_27_), 437.7645 (C_29_), 451.7911 (C_30_), 479.8445 (C_32_), 493.8712 (C_33_) and 507.8978 (C_34_). Additionally, *m/z* 407.6952 was monitored for the presence of the C_27_ mycolipenate carboxylate ion, characteristic of *M*. *tuberculosis* [18].

#### c). Isotopic Analysis

Strontium and oxygen isotopic analysis were carried out on tooth enamel of GC96 to shed light on the individual’s childhood origins.

For strontium isotope analysis, a longitudinal section of enamel approximately 1mm thick was removed from the lower second molar using a hand drill and diamond cutting disk. The sample was cleaned in an ultrasonic bath in 18MΩ H_2_O for ten minutes and dried overnight. For analysis, the sample was mounted in the laser cell by pressing into blue-tack. Sr isotopic analysis was performed on a Finnegan Neptune multi collector ICP-MS with a New Wave 193 nm ArF homogenized excimer laser, using the oxide reduction technique of De Jong [20, 21]. The measurement of Sr isotopes by laser ablation has only recently been made reliable. The primary difficulty has been the molecular interference on ^87^Sr of ^40^Ca^31^P^16^O^+^ which is the primary constituent of the enamel matrix. Other potential problems come from double-charged rare earth elements which give mass-to-charge ratios of between 84 and 88, calcium-calcium and calcium-argide dimers which can interfere with ^84^Sr, ^86^Sr and ^88^Sr, in addition to potential ^87^Rb and ^86^Kr interferences. Our approach has been to minimize oxide formation (monitored as ^254^(UO)^+^/^238^U^+^) through careful control of plasma conditions, and to monitor and reject teeth that have significant rare earth concentrations which we consider diagenetic. We correct for the ^86^Kr using an on peak gas blank and for rubidium interference using the natural ^87^Rb/^85^Rb ratio of 0.385617. A small positive offset from known values ^87^Sr/^86^Sr of standards is usually observed, but is within the precision of a typical measurement.

Time series of strontium isotopes are obtained as continuous data by moving the tooth along the growth axis of the tooth at 5μms^-1^ as the laser pulses with a repetition rate of 10Hz and spot size 150 μm, giving a fluence of c.8.6 Jcm^-2^. Strontium isotopes in the dentine were measured as spot samples by pulsing the laser for 240 s at each spot without moving the sample.

Repeat analysis of an in-house ashed bovine pellet standard (BP1) bracketing analyses of GC96, showed an offset of +89±98 ppm (1 sigma) for the laser ablation analyses over TIMS values. This is within the precision of individual measurements of 150-300ppm and the total variation within the enamel of 1200ppm, and is therefore considered insignificant to our interpretation of the isotopes.

For oxygen isotope analysis a second and similar section was taken from the same tooth. Surface dirt and any dentine were removed from the section using a dental bur. The sample was ground to a fine powder in an agate mortar under acetone, dried and leached for 1 hour in 0.1M acetic acid to remove diagenetic carbonate. The sample was then centrifuged and washed in 18MΩ H_2_O three times and dried.

The oxygen isotopes in the carbonate fraction of tooth enamel were measured on an Isoprime Dual-Inlet mass-spectrometer connected to a Gilson auto-sampler using standard carbonate procedures. For comparison with published measurements on enamel phosphate oxygen isotopes and rainwater δ^18^O, structural carbonate values were converted to phosphate values and then to drinking water equivalents [22, 23].

#### d). Radiocarbon dating

Two radiocarbon samples, taken from the fibula of burial GC96, were submitted to the Scottish Universities Environmental Research Centre (SUERC) and Oxford Radiocarbon Accelerator Unit (ORAU) for radiocarbon dating. The sample processed at SUERC was pre-treated following a modified method [24], converted to carbon dioxide in a pre-cleaned sealed quartz tube [25], graphitised as described by Slota *et al*. [26] and measured by Accelerator Mass Spectrometry (AMS) [27]. The sample dated at ORAU was processed using the gelatinisation and ultrafiltration protocols described by Bronk Ramsey *et al*. [28] and Brock *et al*. [29]. The sample was combusted, graphitised, and dated by Accelerator Mass Spectrometry (AMS) as described by Bronk Ramsey *et al*. [30].

Carbon and nitrogen stable isotope analysis was applied to the samples as the potential for diet-induced radiocarbon offsets if an individual has taken up carbon from a reservoir not in equilibrium with the terrestrial biosphere might have implications for understanding when the individual died [31].

## Results

### 1. Osteology

#### a). Skeletal changes

Based on the cranial and pelvic features, GC96 was sexed as male. The auricular surface, pubic symphysis, complete epiphyseal fusion and light dental wear suggested that the individual was between 25 and 35 years of age at death. Pathological changes were identified on the right ulna, left and right tibiae and fibulae and the left and right foot bones.

On the distal posterior surface of the right ulna, above the styloid process, a small patch (≈1cm^2^) of remodelled periosteal new bone growth was observed.

**Fig 2:**
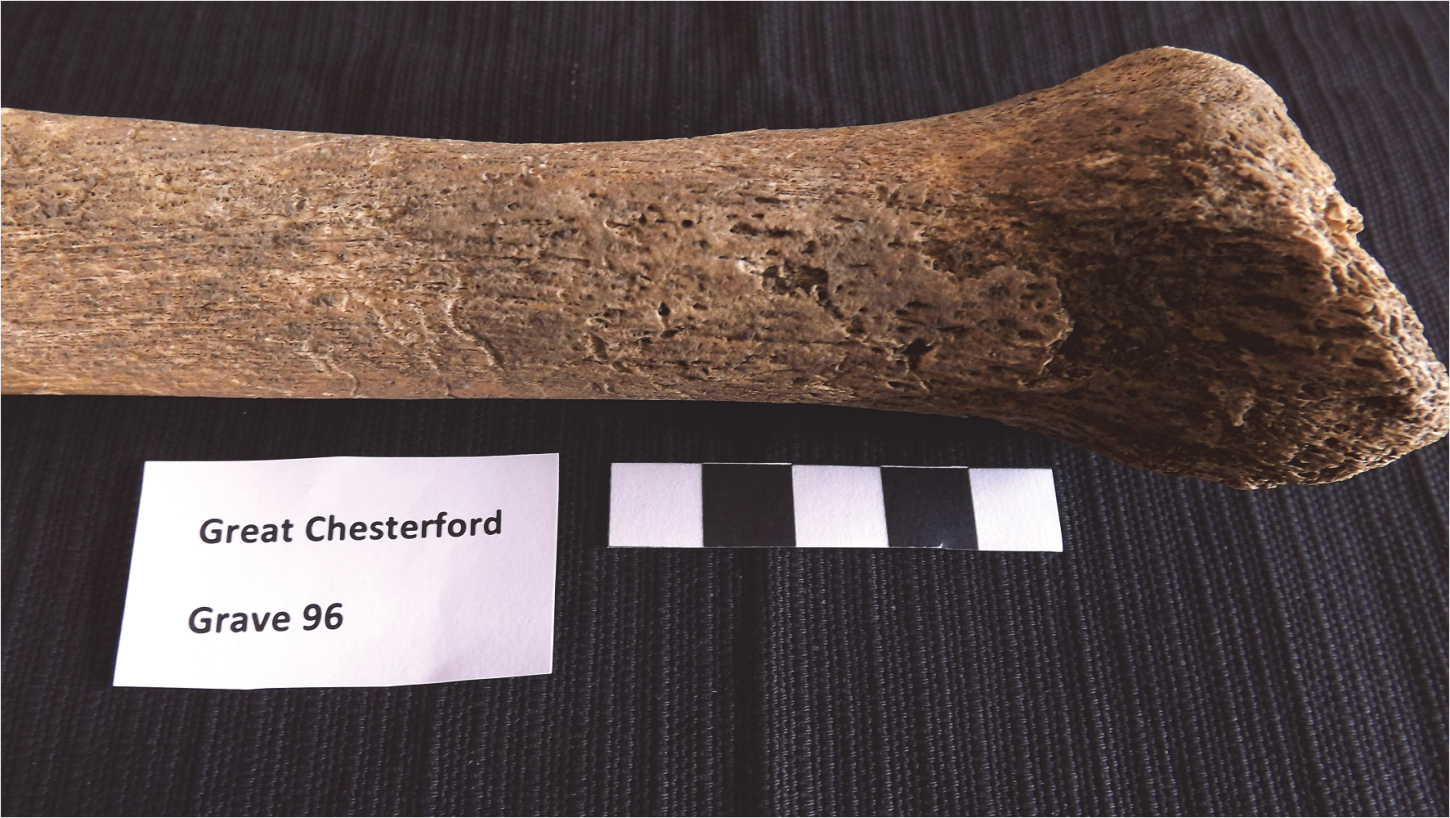
Left tibia of GC96 showing evidence of inflammatory pitting and presence of both woven and remodelled lamellar bone on the subperiosteal shaft.

Both tibiae and fibulae had changes extending from the proximal midshaft onto all of the bone surfaces inferior to it. All four bones had new bone growth on the periosteal surfaces. On both the left and right sides, remodelled lamellar bone was evident, which was orientated longitudinally and took on a nodular appearance where the new bone was thickest, particularly along the interosseous borders. The nodular appearance suggests multiple episodes of new bone apposition with healing. On the left tibia, woven bone, which was porous and disorganised, was observed on the medial malleolus and on the medial and anterior aspect of the distal diaphysis (Fig 2). The nature of the lesions suggests an active process at the time of death. In addition to the new bone growth, increased bone porosity is seen in the distal diaphysis and epiphysis of the left tibia.

**Fig 3:**
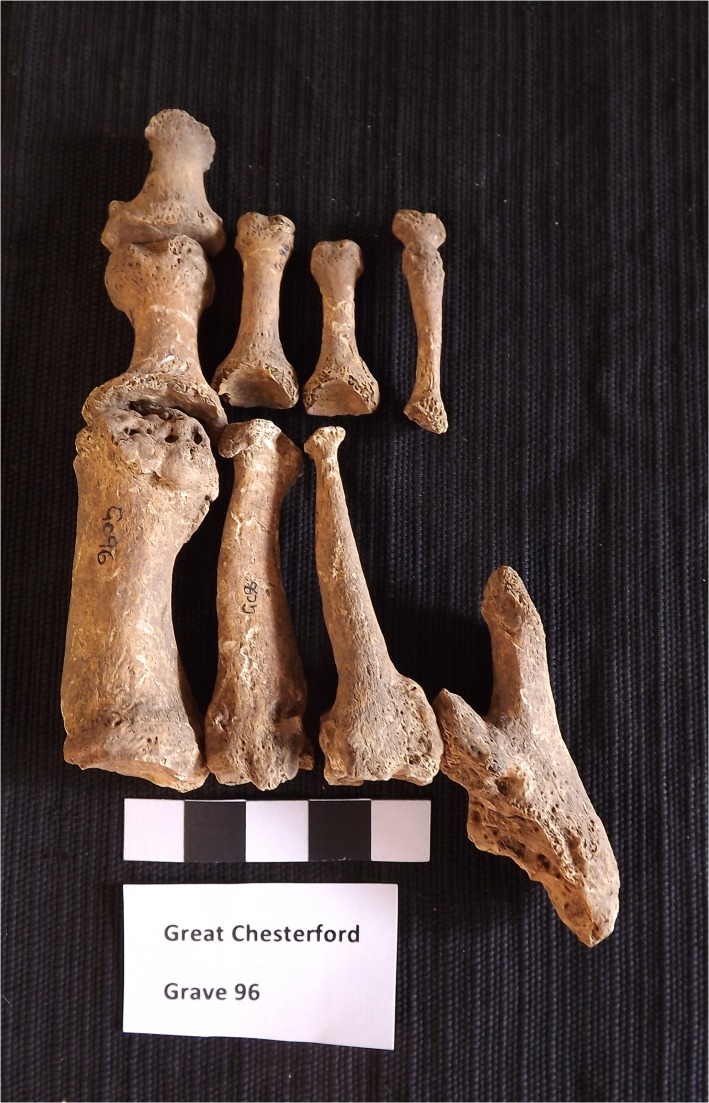
Pencilling of second, third and fifth metatarsals with destructive lesions to the proximal joint surfaces possibly caused by leprosy.

On the right foot, with the exception of right metatarsal one, the tarsometatarsal joints have undergone extensive remodelling, largely as a result of bone destruction. The right second, third and fifth metatarsals have mid-shaft pencilling and almost complete destruction to the distal articular surfaces (Fig 3). It is not clear whether right metatarsal four had been lost *pre*- or *post-mortem*, but a small fragment remains ankylosed to the proximal shaft of metatarsal five. The head of the right first metatarsal and the corresponding proximal joint surface of the hallical phalanx have destructive lesions resembling those at the tarsometatarsal joints. The proximal and intermediate phalanges of the fourth pedal ray are ankylosed. On the left foot, thick new bone growth surrounds the mid-shaft of the left first metatarsal and portions of the left second metatarsal. The sharp edges to the new bone growth and disorganised appearance suggest that the lesion was active at the time of death.

### 2. Biomolecular studies

#### a). Ancient DNA

**Fig 4:**
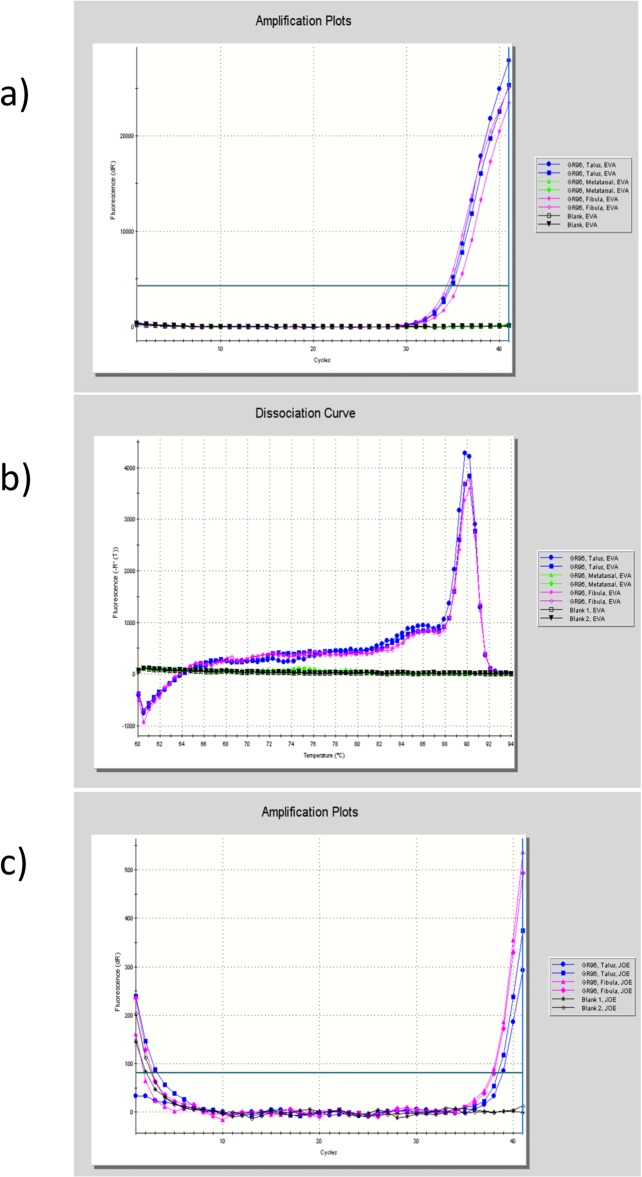
a. RLEP PCR amplification profiles for DNA extracts prepared from the first metatarsal, left talus and left fibula from GC96. Product formation was monitored on an Agilent Mx 3005P qPCR system using the fluorophore EVAGreen. b. Dissociation curve of RLEP PCR products seen in 4a. Note single peaks from talus and fibula at around a melting temperature of 90°C. This is the expected T_melt_ for the RLEP 111 bp amplicon. c. 18-kD PCR amplification profiles for DNA extracts prepared from left talus and left fibula from GC96. Product formation was monitored on an Agilent Mx 3005P qPCR system using a specific hybridization probe.

1. Screening methods. Extracts prepared from the left fibula, left talus and the first metatarsal were all tested with the RLEP and 18-kD antigen screening methods. The left fibula and talus were found to be positive for both markers (Table 1, composite Fig 4A–4C) and these extracts were subsequently used for genotyping. In contrast, reactive bone taken from the first metatarsal was PCR negative using both RLEP assays and the 18-kD method. A second set of samples, taken from left talus, pooled fibulae (L+R) and calcaneum were positive for RLEP (both 111 and 78 bp amplicons) and also for the 18-kD antigen DNA, although the calcaneum was only weakly positive for the latter marker (Table 1). Products from these real-time experiments on the MxPro platform were additionally separated using gel electrophoresis on 3% or 3.5% agarose gels to confirm amplicons of expected size had been produced and this was always found to be the case.
2. SNP typing. Genotyping using PCR and Sanger sequencing of selected informative SNPs, showed that this isolate belonged to the 3I lineage (Table 3). The aDNA findings in GC96 showed consistent molecular behaviour with what is known of extant 3I strains, in that SNP loci 1,133,492 and 7,614 exhibited characteristic bases (both T) and possessed one copy of an eleven base element in indel_17915. Additional subtyping revealed G at position 1,527,056, indicating a 3I-1 strain [15].
3. VNTR typing. We undertook multiple loci variable number tandem repeat (VNTR) analysis (MLVA) of three loci [32]. This showed a 14-6-2 profile for the AGA-20, GTA-9 and 21–3 loci respectively (Table 3). We have not encountered this profile before in ancient cases of LL, which provides additional confirmation of the specific nature of the aDNA evidence obtained from GC96.
4. *Mycobacterium tuberculosis* complex DNA. Selected extracts were shown to be negative for evidence of MTB complex DNA.

**Table 3:** Genotyping data (SNP & VNTR) obtained for burial GC96.

SNP position in TN genome.	GC96 result.	Inference.
14,676	C	
1,642,875	T	
2,935,685	C	Type 3
413,902	G	
591,857	C	
1,133,492	T	Type 3I
2,312,059	C	
7,614	T	Type 3I
1,113,923	A	
1,104,235	G	
3,102,787	C	
Indel_17915 11bp repeat	1 copy	3I-1 or 3I-2
1,527,056	G	**Type 3I-1**
**VNTR loci.**		
AGA(20), ML2345	14 copies	
GTA(9), ML2172-3	6 copies	
21–3, ML0058	2 copies	

#### b). Lipid biomarker analyses

*Mycolic Acids.*

**Fig 5:**
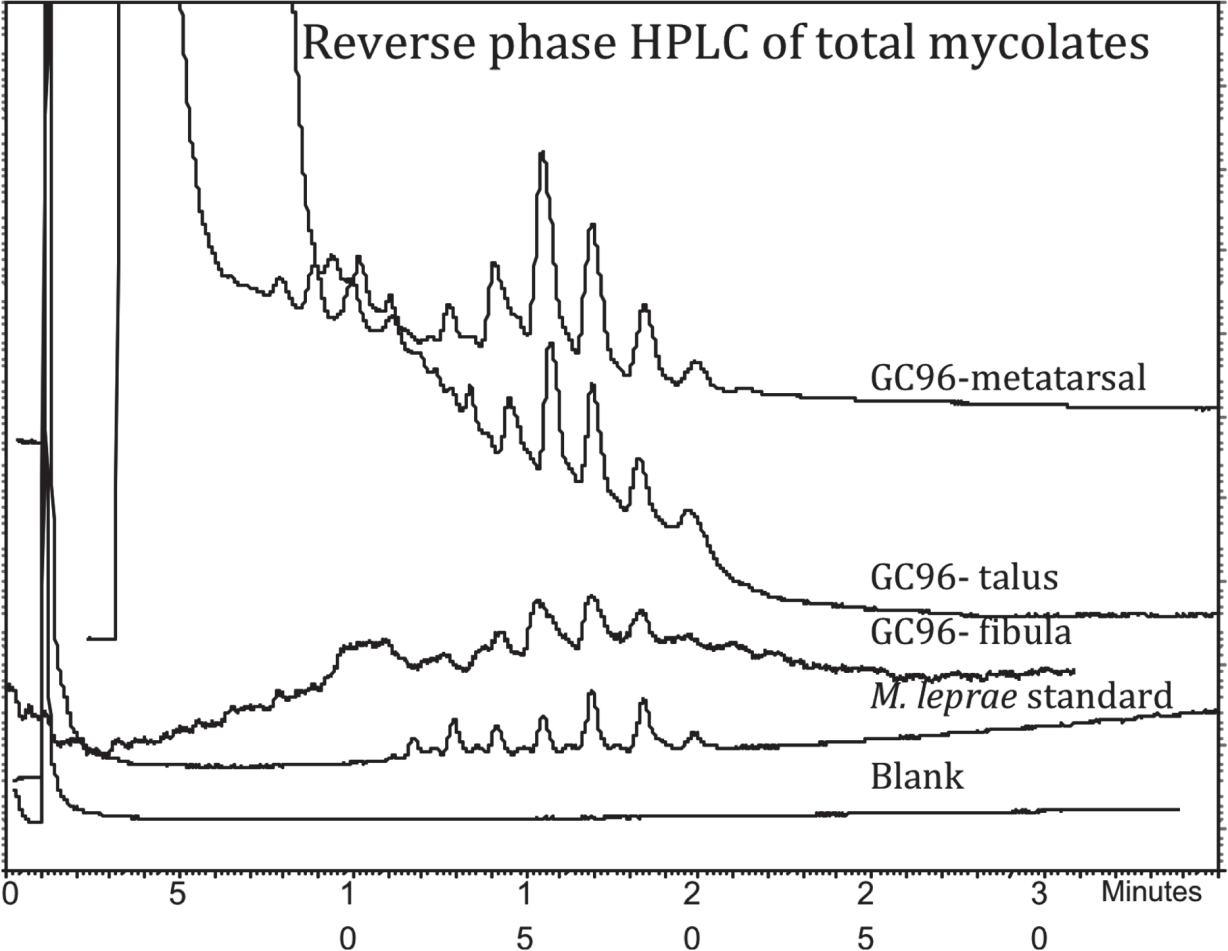
Reverse phase fluorescence HPLC of PBA-PFB derivatives of total mycolic acids from inhumation GC96 metatarsal, talus and fibula extracts.

Reverse phase HPLC of the total PBA-PFB mycolate fractions indicated the presence of mycolic acids in all samples (Fig 5). The profile was strongest in the sample of reactive bone scraped from the metatarsal, closely followed by that from the talus; the weakest profile was obtained from bone taken from the fibula end. None of the traces corresponded exactly with the standard profile for extracts of *M*. *leprae*. Additional information was sought by sequential normal and reverse phase HPLC of total mycolates, as shown in (S1 Fig). In essence, the normal phase analysis of collected MAs, from the initial reverse phase separation (Fig 5 and S1A Fig), showed principally the presence of components corresponding to α-mycolates (S1B Fig). The reverse phase separation of the collected α-mycolates indicated homologues, with a main C_76_ component (S1C Fig). Reverse phase HPLC (S1D Fig and S1E Fig) of the minor fractions from normal phase separations (S1B Fig) gave no peaks of great significance.

##### Mycocerosic Acids

**Fig 6:**
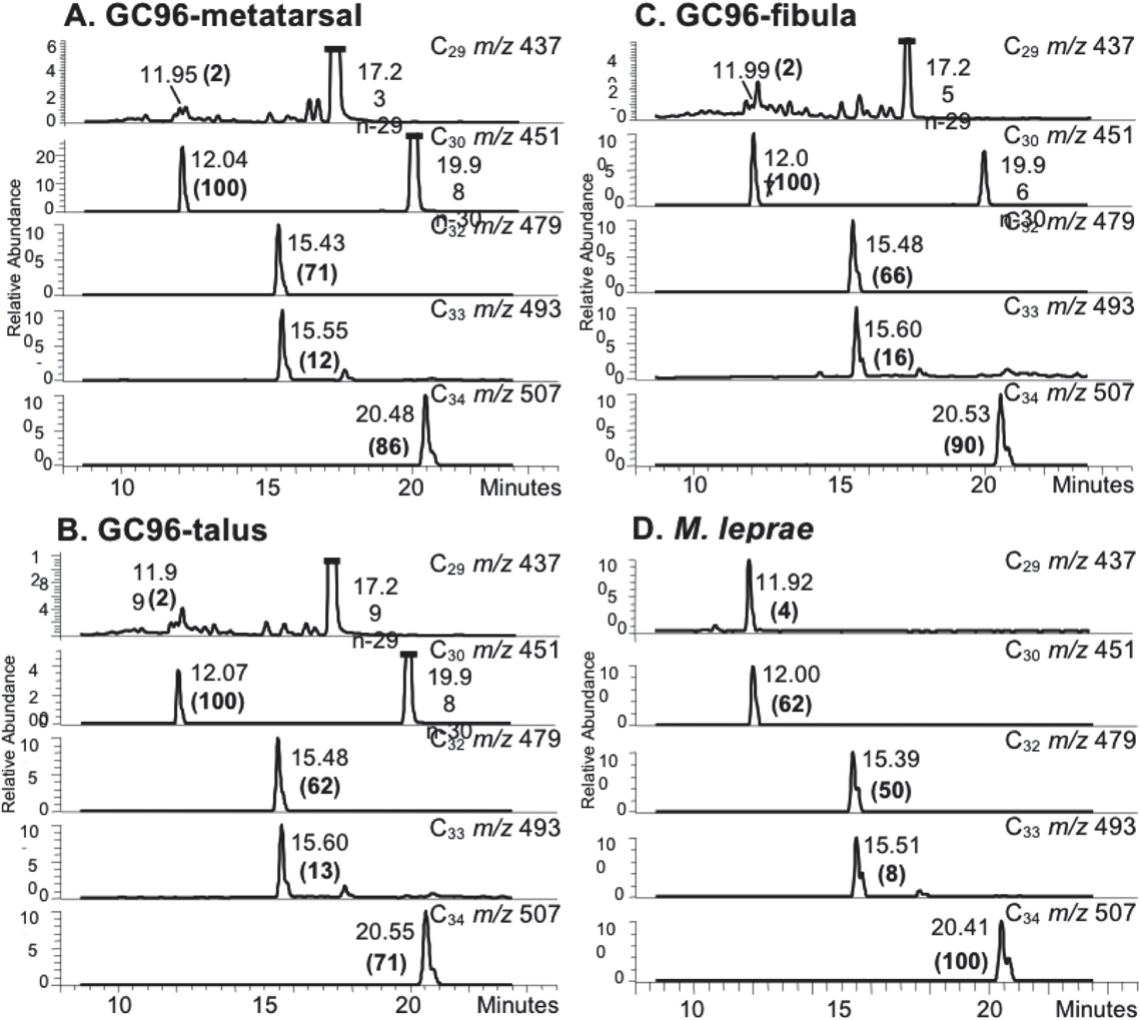
Mycocerosic acid profiles from inhumation GC96 metatarsal, talus and fibula extracts. Profiles are for selected ion monitoring NI-CI GC-MS of mycocerosic acid pentafluorobenzyl esters extracted from metatarsal (**A**), talus (**B**) and fibula (**C**), compared with *M*. *leprae* standard (**D**). Negative ions at *m/z* 437, 451, 479, 493 and 507 are for carboxylates from C_29_, C_30_, C_32_, C_33_ and C_34_ mycocerosates. The intensities of the mycocerosate peaks, in brackets, are normalized to that (100) of the major C_30_ mycocerosate in the GC96 profiles and major C_34_ mycocerosate for standard *M*. *leprae*. In the GC96 *m/z* 437 profiles, the peaks with retention times 17.23, 17.25 and 17.29 correspond to a 29 carbon straight-chain acid (n-29); in the *m/z* 451 profiles, peaks at 19.96 and 19.98 min correspond to a 30 carbon straight-chain acid (n-30).

All extracts showed the presence of C_29_, C_30_, C_32_, C_33_ and C_34_ mycocerosates, correlating well with the standard profile for *M*. *leprae* (Fig 6); no C_27_ mycocerosate or C_27_ mycolipenate was detected. Partial racemisation of mycocerosates during the alkaline hydrolysis leads to the formation of diasteroisomers, which resolve on gas chromatography to give characteristic doublets [18, 33].

###### c). Isotope analysis

**Fig 7:**
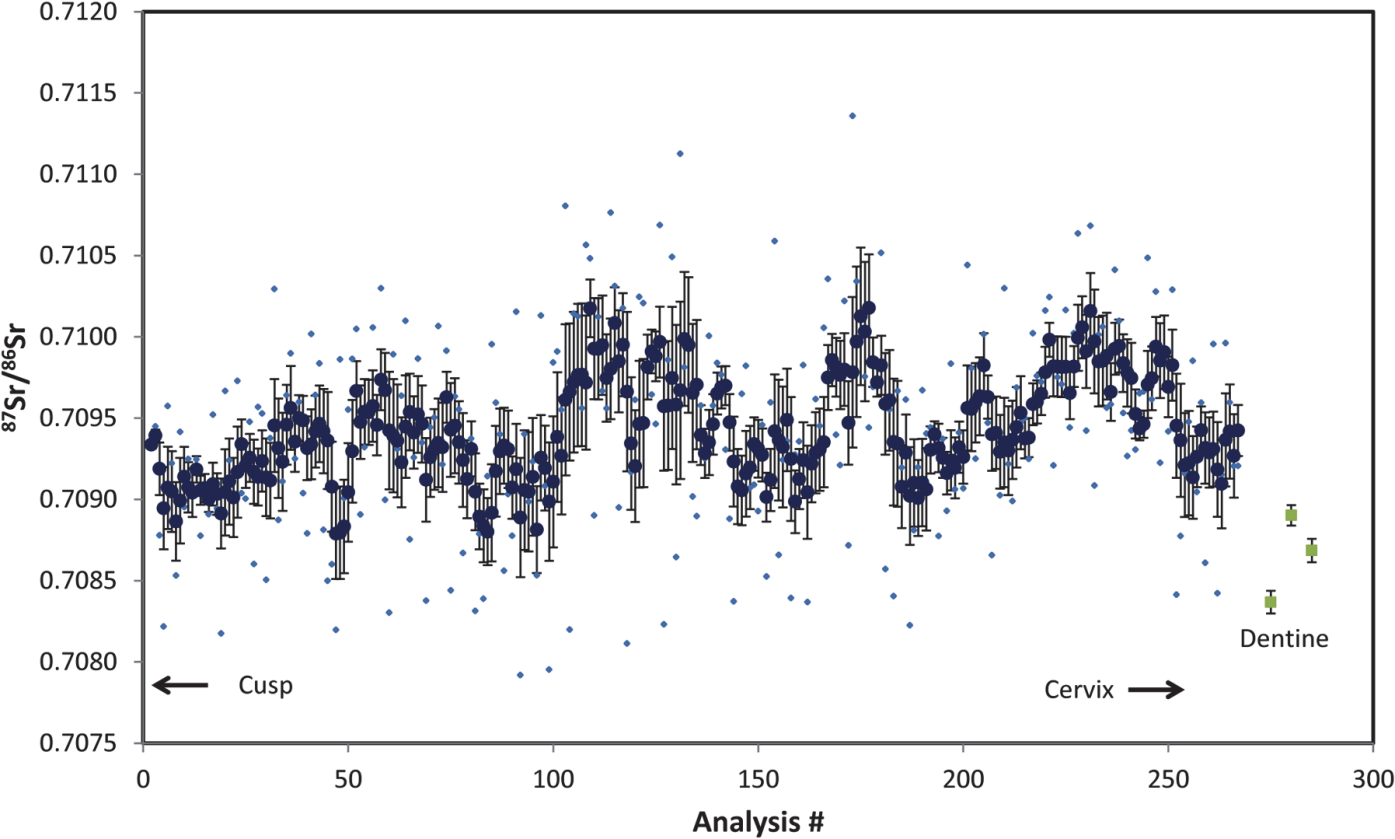
Laser ablation Sr isotopic analysis of GC96 lower M2 enamel. The analyses are sequential from the cusp to the cervix of the crown. Light coloured points represent individual measurements and darker points represent the 5 point moving mean of the measurements with error bars representing the standard error on this mean. Also shown are the three dentine spot analyses.

The strontium analysis of the enamel showed periodic variation in ^87^Sr/^86^Sr between 0.7088 and 0.7101 (Fig 7). The bedrock geology in the immediate vicinity to Great Chesterford is Upper Cretaceous chalk, which would give a ^87^Sr/^86^Sr of 0.7073–0.7080 [34]. This range is not represented in the enamel analyses (Fig 7), but is reflected in the lower isotopic ratios of the dentine which reflects a mix of biogenic strontium (i.e. similar values to the enamel) and diagenetic strontium from the immediate burial environment (presumably in the range of 0.7073–0.7080). On face value then, the strontium results suggest this individual is not local to Great Chesterford. However, strontium isotopic values of 0.7088–0.7101 are typical for large swathes of SE Britain [35 (see map)] including regions abutting the Cretaceous Chalk within a few 10s of km of Great Chesterford, so the enamel isotopic range is consistent with a childhood origin somewhere in the wider region.

However, strontium isotopic values are rarely unique for a specific location, and the oxygen isotope evidence provides stronger evidence for the non-local origin of GC96. The equivalent drinking water, δO^18^ = -8.1 ‰ (Table 4) gives an isotopic value at the extreme range of rainfall values for Britain e.g. see map [36], with values of >-8.0 **‰** estimated only for NE Scotland (i.e. Grampian and Tayside) and the region between the coast and the Eastern edge of the Pennines. Values between -7.0 to -8.0**‰** are estimated for East Anglia, and individuals from Great Chesterford should be closer to -7.5‰ [37]. Therefore, the equivalent drinking water estimates may suggest a childhood origin in NE Scotland, the Eastern edge of the Pennines or further East in continental Europe. The older geology of NE Scotland is incompatible with the measured Sr isotopes, and can be ruled out. Maps based on modern rainwater δO^18^_,_ show values of -8 to -9**‰** representing an approximately North-South strip of Europe running through Eastern France, Central Germany, Eastern Denmark, and small areas of non-coastal SW Norway [38]. Few of these areas have detailed Sr isotopic maps, but Sr isotopes from medieval burials in SW Norway show ^87^Sr/^86^Sr consistently >0.710 and as high as 0.730 making this area unlikely as an origin for GC96. In contrast, the well-characterized Sr geochemisty of Denmark [39] shows ^87^Sr/^86^Sr in surface waters for most regions of Denmark predominantly in the range 0.708–0.710, consistent with the values obtained for GC96. Sr analyses for Eastern France and Central Germany are not detailed enough to rule out an origin in either, and geologies certainly exist that would give isotopic values compatible with GC96.

**Table 4:** Sr isotopic and δ^18^O values for enamel and dentine sample for GC96.

Sample	87Sr/86Sr	δO^18^ carbonate, pdb, ‰	δO^18^ phosphate equivalent, vsmow, ‰	δO^18^ drinking water equivalent, vsmow, ‰
GC96M2 enamel		-5.25	16.6	-8.1
GC96M2 dentine1	0.708368 ± 0.000070			
GC96M2 dentine2	0.708902 ± 0.000064			
GC96M2 dentine3	0.708685 ± 0.000072			

The structural carbonate fraction was measured and converted to a phosphate equivalent and then to an equivalent drinking water value using Chenery et al. [22]. Typical uncertainty on the δ^18^O carbonate is ±0.15 ‰, 1 sigma.

However, the conversion of measured δO^18^ to equivalent drinking water is complicated by large regression uncertainties (estimated at ±1.0‰, 2σ -) [22], so comparing isotopic measurement on teeth to rainwater is less than ideal and we should be careful not to over-interpret comparisons of δO^18^ in rainwater with equivalent drinking water values. However, the uncertainties on the conversion of δO^18^_carbonate_ to δO^18^_phosphate_ are considerably smaller; Chenery *et al*. [22] estimate a maximum uncertainty of ±0.56‰ (2σ), allowing more a precise comparison between our estimated δO^18^_phosphate_ values to those measured directly on tooth enamel phosphate. Evans *et al*. [40] give δO^18^ phosphate values measured on 615 archaeological teeth on individuals of a presumed British origin. Of these only 39 have δO^18^ phosphate < = 16.6‰, or c.6% of individuals. Taking the maximum uncertainty on the carbonate to phosphate δO^18^ conversion into account, 173 (28%) individuals have δO^18^ phosphate < = 17.2‰. So assuming Evans’ *et al*. analyses are representative of all British δO^18^ values, the likelihood of GC96 being local to Britain lie between 28% and 6%, i.e. GC96 is most likely from somewhere on mainland Europe.

#### d). Radiocarbon dating

The radiocarbon dating results are shown in Table 5. The calibrated dating revealed a range between AD 415–545, which slightly refines the AD 5^th^ -7^th^ century range for when the Anglo-Saxon cemetery at Great Chesterford was believed to have been in use [5]. The calibrated date is relatively imprecise because the age of the sample falls on a slight plateau in the calibration curve (not shown).

**Table 5:** Radiocarbon dating results.

Laboratory number	Sample reference	Material	δ^13^C(‰)	δ^15^N (‰)	C/N ratio	Radiocarbon Age (BP)	Weighted mean	Calibrated Date (95% confidence)
SUERC-49551	GC96 sample B	Fibula	-20-5	11.1	3.3	1526±30	1573±19 BP (T’ = 4.0; n = 1; T’(1%) = 6.0)	cal AD 415–545
OxA-29151	GC96 Sample B	As SUERC-49551	-20.2	10.7	3.3	1603±25	1573±19 BP (T’ = 4.0; n = 1; T’(1%) = 6.0)	cal AD 415–545

The stable isotope results (Table 5) indicate that the individual consumed a diet predominantly based upon temperate terrestrial C3 foods [41, 42]. The radiocarbon results are therefore unlikely to be affected by any significant reservoir effects [43] and the calibrated date range can be regarded as an accurate estimate of the age of the sample.

## Discussion

### 1. The pathological lesions

A number of conditions potentially responsible for the changes observed in GC96 are considered. Erosive arthopathies (EA) cause destruction of joints and the bones of the hands and feet are frequently affected. In particular, seronegative spondyloarthropathies may result in cup and peg deformities, ankylosis of inter-phalangeal joints, and subperiosteal proliferation in the foot and lower leg bones [44, 45]. However, new bone growth on the tibiae and fibulae is usually focused on soft tissue attachment areas [46], a pattern not observed in GC96. In addition, in seronegative spondyloarthropathy, other areas of the skeleton should be diseased, especially the vertebral and sacro-iliac joints [45]. Although the vertebrae were absent, the sacro-iliac joints in GC96 appeared normal.

A consequence of diabetes is neuropathy, which can cause atrophy in the foot bones [47]. Of importance here is the concentric remodelling to the distal metatarsals and phalanges. Additionally, the loss of sensation and impaired circulation in diabetes favors infection in the foot. However prolonged survival with diabetes is unlikely in antiquity [48]. In addition, the age of onset for diabetes-related foot changes is usually over 40 years [49], so the young age of GC96 would also argue against this diagnosis.

The destructive lesions of the metatarsals and phalanges, particularly the blade like deformity of the fifth metatarsal, the ankylosis of pedal phalanges and the form and distribution of the subperiosteal new bone proliferation on the tibiae and fibulae are typical of leprosy [47, 50]. Although leprosy appears to be the most likely cause of the osteological lesions, the absence of the facial skeleton meant that the presence or otherwise of *facies leprosa*, could not be confirmed, so it was difficult to be certain of diagnosis. Hence biomolecular analysis was required to confirm the presence of the disease.

### 2. Ancient DNA

Comparison of leprosy genomes from diverse geographical regions and bioinformatics have led to the development of epidemiological tools, for both investigation of short-term transmission by family members as well as discerning global dissemination of leprosy in prehistory.

Examination of single nucleotide polymorphisms (SNPs) in strains recovered from regions where the disease is still endemic, showed that present-day cases of leprosy are derived from a clone of *M*. *leprae* which probably originated in East Africa and spread with human movements around the world [13]. Using phylogenetically informative SNP polymorphisms from regions where the disease is still endemic, these workers firstly identified four main genotypes which were later extended into 16 subtypes, designated A to O [14]. Type 1 strains, belonging on the first branch, may be subdivided into isolates A-D, type 2 or second branch strains into E-H, third branch into I-M and lastly, fourth branch isolates into N and O strains. The latter show variation in two insertion/deletion loci (indels) and four homopolymeric tracts (HPT) rather than in SNP variability. Truman and co-workers [15] added yet more detail, showing variation within 3I strains in southern states of North America which could be categorized as either 3I-1 or 3I-2, depending on the nucleotide at position 1,527,026 (G in 3I-1 and C in 3I-2) and the number of copies of an 11 bp element within a region known as indel_17915.

Bioarchaeological studies have begun to investigate the strains of *M*. *leprae* responsible for the disease in the medieval period when leprosy was at its height in the UK and continental Europe. Retrieval of near complete genomes of *M*. *leprae* from the early medieval period has provided evidence for ancestors of two of the above lineages in human remains, namely 2F and 3I-1 [3]. These studies have also found that some isolates previously recognised as type 3K more properly belong on a separate branch, now termed branch 0. The earlier phylogenetic model for global dissemination of the four main types and their interrelationship has therefore undergone some refinement in the light of findings from past outbreaks and more geographically separated modern samples. Type 3 isolates have emerged as being of particular interest as they are known to have been responsible for archaeological cases described in central Europe (Hungary, 3K, 3M), north Africa (genotype 3) as well as central Asia (Uzbekistan, 3L) and western Asia (Turkey, 3K). The refined phylogeny of *M*. *leprae* indicates parallel evolution of the five main branches diverging from a common ancestor at some point estimated to be around 2,500 years ago [3] rather than as a result of serial progression of types 1 to 4 by the most parsimonious route of single nucleotide polymorphism. Further work is needed to clarify the relationship between the type 3 strains and the recently identified branch 0, which includes at least some of the type 3K isolates.

The subject of the present study, GC96, was infected with a strain belonging on the 3I lineage. Modern 3I-1 isolates display T and G bases at nucleotide positions 7,614 and 1,113,926 respectively. In GC96, the SNPs were T and A respectively. This appears to be an intermediate genotype between what would be expected from other type 3 strains (C and A) and implies that this individual was infected with a strain which may have been ancestral to modern 3I exemplars. The strain type has little bearing on the pathogenesis or severity of disease, as this is dictated by the immune response to *M*. *leprae*, but rather it may be helpful in understanding the origin of disease in the Anglo-Saxon period. Other type 3I cases have been reported from medieval Britain (Winchester and Ipswich), Denmark and Sweden [2, 51]. A Scandinavian origin for this lineage is therefore one possibility, given the proximity of the Anglo-Saxon tribal homelands in Northern Germany with Denmark. While the oxygen and strontium isotopic results can only give a broad and often ambiguous indication of childhood origin, the phosphate converted oxygen isotopes support a probable non-British origin for GC96, and albeit with a larger uncertainty, the drinking water equivalent values correspond to rainfall in parts of Germany and Denmark, though we cannot rule out with certainty an origin in France, or even a relatively small area of east central Britain.

The 3I lineage is still found in southern states of the USA and was likely taken to the New World by early European settlers. Although the total evidence from the medieval period is limited to around a score of cases, it does seem that this genotype was one of two predominant lineages associated with the rise in disease in Britain at this time. There is also evidence to suggest that the 3I lineage was present in Britain much later in the timeline of European leprosy, which had begun to decline by this time [52]. We have previously reported this lineage in a case from 13^th^-16^th^ century Ipswich, Suffolk, UK [53]. Given persistence of the 3I lineage over nearly 800 years, it seems unlikely that a change in genetic make-up of the bacillus was responsible for the decline in European leprosy. This was supported by comparison of present day 3I whole genomes with those recovered from both Winchester and Scandinavia [3] which revealed remarkably high degrees of conservation amongst the ancient and modern strains.

### 3. Lipid Biomarker Analysis

Excellent mycocerosate profiles, typical of *M*. *leprae* (Fig 6), were conclusive confirmation for the presence of leprosy in GC96. In particular, major amounts of C_34_ mycocerosate, accompanied by lesser proportions of C_33_ mycocerosate, are decisive leprosy biomarkers [54]. A particular diagnostic feature is the chromatographic overlap of the pentamethyl-branched C_33_ mycocerosate with the tetramethyl-branched C_32_ mycocerosate. The lack of any C_27_ mycolipenate correlates with the absence of tuberculosis infection [18, 33]. The excellent mycocerosate profiles (Fig 6) support earlier suggestions [18, 55] that the very hydrophobic phthiocerol dimycocerosate waxes are exceptionally stable, with the potential to persist far back into antiquity.

Mycolic acid profiles (Fig 5; S1 Fig) were not so decisive, with evidence for considerable degradation. The initial reverse phase HPLC profiles of total mycolates (Fig 5; S1A Fig) were clear and strong for the metatarsal extract, but progressively weaker for the talus and fibula samples. The positions of the peaks were consistent across all three specimens, but they did not correspond precisely to those for modern standard *M*. *leprae* (Fig 5). Normal phase HPLC allows separation of the individual classes of mycolic acids, which in the case of *M*. *leprae* are the so-called α-mycolates and ketomycolates [2,19]; methoxymycolates, characteristic of *M*. *tuberculosis*, are absent. In the present examples, normal phase HPLC (S1B Fig) gave clear peaks only for α-mycolates. Particularly, in the case of the talus extract, it was possible to discern two minor peaks, eluting after the α-mycolates (S1B Fig), so fractions of these potential biomarkers were collected from all three normal phase separations for reverse phase HPLC investigation. The α-mycolate class gave good clean reverse phase profiles for the metatarsal and talus extracts, with a weak trace for the fibula (S1C Fig). Significantly, the extracted α-mycolates from GC 96 are two carbons shorter than those of standard *M*. *leprae*. Since clean profiles are recorded, the α-mycolates would not appear to have suffered extensive diagenetic degradation, so an explanation must await the analysis of a greater number of archaeological samples.

Insignificant traces are recorded for components corresponding to ketomycolates (S1E Fig), suggesting that any such mycolates have been degraded beyond recognition. Selective diagenetic degradation of ketomycolates has been previously observed in ancient leprosy skeletons (Sk7, Sk19) from Winchester [2] and other tuberculosis and leprosy cases [55]. As noted above, methoxymycolates have not been characterized from *M*. *leprae*, but in a previous study [2] reverse phase HPLC analysis of fractions eluting in the region expected for methoxymycolates in normal phase HPLC separations, showed the presence of minor components of unknown significance in two skeletons (Sk7, Sk8). Again, in the present study, extracts of the metatarsal and talus revealed the presence of two very weak peaks (S1D Fig). Further studies will be required to determine if such minor components have biomarker potential, but it is important not to overlook their presence. Indeed, in standard *M*. *leprae* the presence of some very minor components is indicated, but these do not correspond to those in the metatarsal and talus specimens (S1D Fig).

The Great Chesterford case is thus of particular interest in understanding the origins of leprosy in the British Isles, being one of the earliest radiocarbon dated cases with supportive DNA evidence and genotyping of the isolate. The earliest purported example of leprosy from Britain that has been described in the published literature dates from the 3^rd^-4^th^ century AD and comes from Poundbury, Dorset [56]. This burial comprises lower leg and foot bones only. Although these show changes that are compatible with leprosy, the presence or otherwise of the more firmly diagnostic facial changes could not be ascertained, so the diagnosis is controversial [57]. The first cases of leprosy in Britain showing diagnostic facial signs date from the early Anglo-Saxon period (Table 6).

**Table 6:** Early Anglo-Saxon (5^th^-7^th^ century) cases of leprosy from Britain showing rhino-maxillary changes.

Site	Burial	Date	Sex & age	Reference
Barrington Edix Hill, Cambridgeshire.	Grave 18 Sk42B	AD575-650	M 17–25	Duhig 1999 [58].
Broughton Lodge, Nottinghamshire	62/63/64E	AD475-600	U 50+	Roberts 1993 [59].
Burwell, Cambridgeshire	Grave 111	7^th^ cent. AD	M Adult	Møller-Christensen & Hughes 1962 [60].
Cannington, Somerset	Burial 159	5^th^ cent. AD?	F ca. 22	Brothwell et al. 2000 [61].
Dunstable Marina Drive	Burial E1	AD650-670	J 7–8	Beavan & Mays, 2013 [62].
Eccles, Kent.	-	7^th^ cent. AD	M 25–30	Manchester 1981[63].
Great Chesterford, Essex	GC96	AD415-545	M 15–20	Present study.
Tean, Scilly Isles	Burial IV	7^th^-8^th^ cent. AD	M Adult	Brothwell 1961 [8].

Sex: M = adult male, F = adult female, U = unsexed adult, J = juvenile. Age, in years. All dates, save Dunstable Marina Drive and Great Chesterford are based on artifact typologies or other archaeological criteria.

If it is accepted that rhinomaxillary changes of the type described by Møller-Christensen [64] and Andersen & Manchester [65], or else confirmation of the presence of *M*. *leprae* using ancient DNA, or other biomarkers, are required for a secure diagnosis, then there are few cases of leprosy worldwide that predate the Great Chesterford example. The earliest such cases come from 2^nd^ century BC Egypt [66]. In western Asia there is an example from 1^st^ century AD Israel [67], 1^st^-4^th^ century AD Uzbekistan [68] and 4^th^-7^th^ century AD Bet Guvrin, Israel [69]. Cases from continental Europe are known from Italy from the 2^nd^-3^rd^ century AD [70] and the 5^th^ century AD [71] and, north of the Alps, from Halland, Sweden (AD70-560) [72] and Lauchheim, Baden-Württemberg, Germany (AD450-680) [73], and a very early case (4^th^ millennium BC) has recently been suggested from Hungary [74]. Once more European and Middle Eastern archaeological cases are genotyped, the results of this research will be important for understanding the spread of leprosy and any significant relationship that might exist between European and Middle Eastern strains.

## Conclusions

Pathological lesions of a 5^th^ – 6^th^ century male from Great Chesterford, Essex (GC96), were compatible with, but not pathognomonic of leprosy. Subsequent DNA and lipid biomarker analysis confirmed the presence of *M*. *leprae*. Additionally, genotyping showed GC96 to have a strain of the 3I lineage, but likely ancestral to present day exemplars. The 3I lineage is associated with LL cases in Medieval England, Denmark and Sweden, but also later emerged in the Americas, where it persists in southern states. As Sr and O stable isotope ratios suggest that GC96 was likely to be non-local to the region, the evidence suggests that this lineage could have been bought to England as early as the 5^th^ century. Accordingly, considering the early medieval population movements in north Western Europe at this time, this research potentially supports the notion of a Scandinavian origin for the 3I strain which emerged in Britain. This multidisciplinary research adds considerable detail to the evolution of *M*. *leprae* and helps us begin to piece together the possible rise in prevalence of leprosy in the context of population movements in the Anglo-Saxon period.

## Supporting Information

S1 Fig Gas chromatography mass spectrometry. Negative ion chemical ionization gas chromatography mass spectrometry (NICI-GCMS) was carried out at Swansea University. The instruments were a Thermo Scientific DSQII Mass Spectrometer coupled to a Thermo Scientific TRACE GC Ultra gas chromatograph. A Phenomenex Zebron ZB-5 (5% phenyl, 95% dimethylpolysiloxane) capillary column (30 m × 0.25 mm i.d. × 0.25 μm film thickness), using He as carrier gas (constant flow mode 1.2 ml min-1) and ammonia as the CI reagent gas, was used. An initial GC oven temperature of 200°C was increased to 300°C at a gradient of 17.5°C min^-1^, the final temperature being held for 17.5 min. The ion source temperature was 170°C. The injector used was a programmable temperature vapourising injector, which started at 50°C for 0.2 min and increased to 300°C at a rate of 10°C s^-1^ where it stayed for 0.5 min. S1 Fig. Complete HPLC analysis of PBA-PFB derivatives of mycolic acids from extracts of GC96 metatarsal, talus and fibula and standard *M*. *leprae*. A. Reverse phase HPLC of total mycolates; B. Normal phase HPLC of total mycolates, collected from reverse phase separation A; C. Reverse phase HPLC of α-mycolates, collected from normal phase separation B; D. Reverse phase HPLC of fraction corresponding to “methoxymycolates”, collected from normal phase separation B, E. Reverse phase HPLC of ketomycolate fraction, collected from normal phase separation B.
(TIF)
