# Supplementary figures and images for: Osteological, Biomolecular and Geochemical Examination of an Early Anglo-Saxon Case of Lepromatous Leprosy
Source: PLoS One. 2015 May 13;10(5):e0124282. doi: 10.1371/journal.pone.0124282 (PMC4430215; doi:10.1371/journal.pone.0124282)

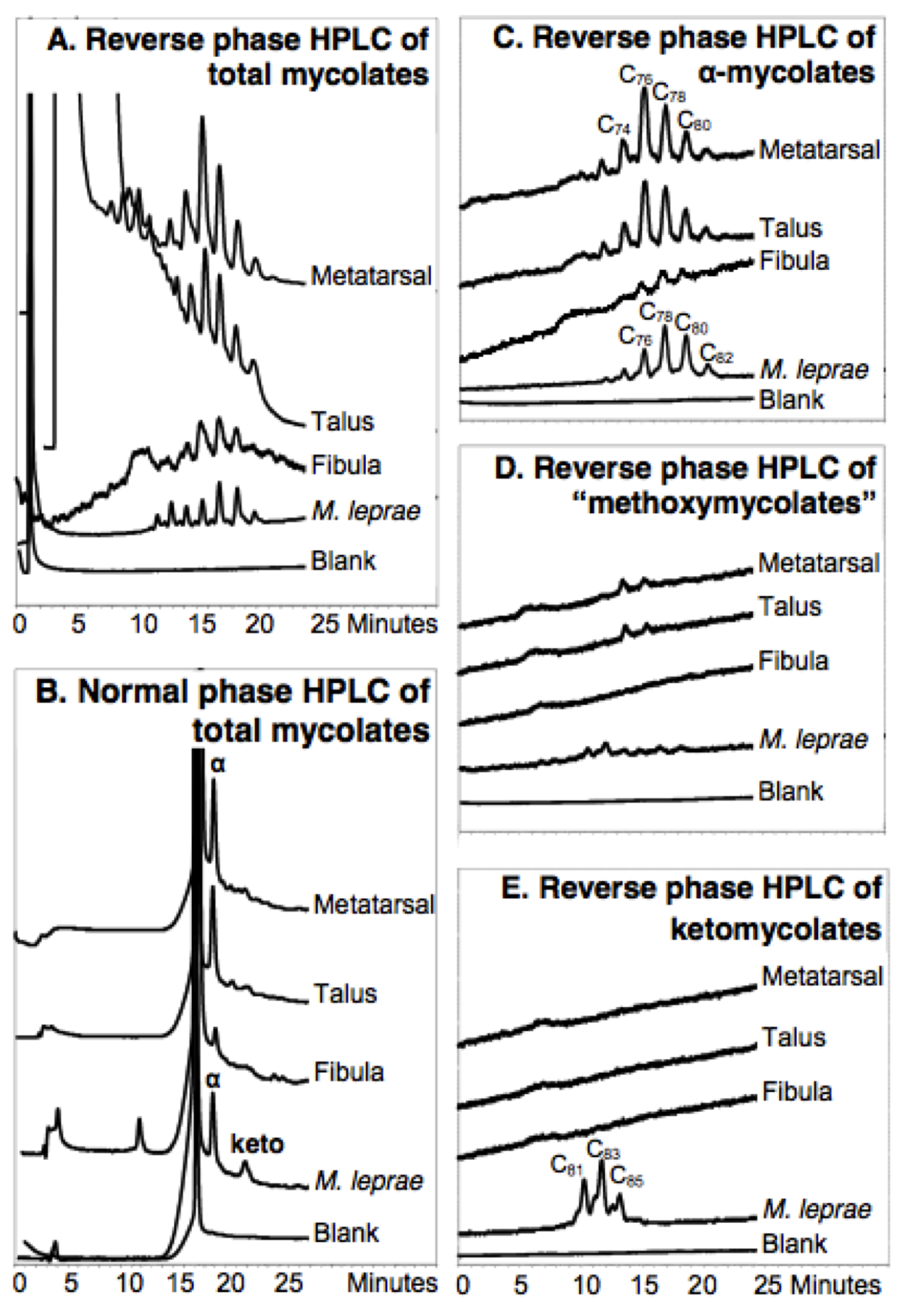

Supplement: S1 Fig — Negative ion chemical ionization gas chromatography mass spectrometry (NICI-GCMS) was carried out at Swansea University. The instruments were a Thermo Scientific DSQII Mass Spectrometer coupled to a Thermo Scientific TRACE GC Ultra gas chromatograph. A Phenomenex Zebron ZB-5 (5% phenyl, 95% dimethylpolysiloxane) capillary column (30 m × 0.25 mm i.d. × 0.25 μm film thickness), using He as carrier gas (constant flow mode 1.2 ml min-1) and ammonia as the CI reagent gas, was used. An initial GC oven temperature of 200°C was increased to 300°C at a gradient of 17.5°C min-1, the final temperature being held for 17.5 min. The ion source temperature was 170°C. The injector used was a programmable temperature vapourising injector, which started at 50°C for 0.2 min and increased to 300°C at a rate of 10°C s-1 where it stayed for 0.5 min. S1 Fig. Complete HPLC analysis of PBA-PFB derivatives of mycolic acids from extracts of GC96 metatarsal, talus and fibula and standard M. leprae. A. Reverse phase HPLC of total mycolates; B. Normal phase HPLC of total mycolates, collected from reverse phase separation A; C. Reverse phase HPLC of α-mycolates, collected from normal phase separation B; D. Reverse phase HPLC of fraction corresponding to “methoxymycolates”, collected from normal phase separation B, E. Reverse phase HPLC of ketomycolate fraction, collected from normal phase separation B. (TIF) [file pone.0124282.s001.tif]
